# Effects of a Combination of *Berberis aristata*, *Silybum marianum* and Monacolin on Lipid Profile in Subjects at Low Cardiovascular Risk; A Double-Blind, Randomized, Placebo-Controlled Trial

**DOI:** 10.3390/ijms18020343

**Published:** 2017-02-07

**Authors:** Derosa Giuseppe, D’Angelo Angela, Romano Davide, Maffioli Pamela

**Affiliations:** 1 Centre of Diabetes and Metabolic Diseases, Department of Internal Medicine and Therapeutics, University of Pavia and Fondazione IRCCS Policlinico San Matteo, 27100 Pavia, Italy; labmedmol@smatteo.pv.it (D.A.); dr.davideromano85@gmail.com (R.D.); pamelamaffioli@hotmail.it (M.P.); 2 Center for the Study of Endocrine-Metabolic Pathophysiology and Clinical Research, University of Pavia, 27100 Pavia, Italy; 3 Laboratory of Molecular Medicine, University of Pavia, 27100 Pavia, Italy; 4 Center for Prevention, Surveillance, Diagnosis and Treatment of Rare Diseases, Fondazione IRCCS Policlinico San Matteo, 27100 Pavia, Italy

**Keywords:** *Berberis aristata*, lipid profile, monacolin K, nutraceutical, *Silybum marianum*

## Abstract

The aim of this study was to evaluate the efficacy and safety of an anti-hypercholesterolemic agent containing *Berberis aristata*, *Silybum marianum* and monacolin K and KA in a sample of Caucasian patients at low cardiovascular risk according to Framingham score. The primary outcome was to evaluate the effects of this nutraceutical combination on lipid profile; the secondary outcome was to evaluate the effect on some inflammatory markers, in particular high sensitivity C-reactive protein and tumor necrosis factor-α interleukin-6. One hundred and forty-three patients were randomized to placebo or Berberol^®^ K, once a day, during the dinner, for 3 months, in a randomized, double-blind, placebo-controlled trial. We recorded a significant reduction of fasting plasma glucose with Berberol^®^ K compared to placebo (−12.2%, *p* < 0.05). Moreover, we recorded an increase of fasting plasma insulin with Berberol^®^ K both compared to baseline and to placebo (+9.9%, *p* < 0.05). Accordingly, the homeostasis model assessment (HOMA) index obtained after treatment with Berberol^®^ K was lower than the one in the placebo group (−2.8%, *p* < 0.05). No variations of lipid profile were observed with placebo, while there was a significant decrease of total cholesterol (−20.5%, *p* < 0.05), triglycerides (−17.7%, *p* < 0.05), and low density lipoprotein (LDL) cholestero (−27.8%, *p* < 0.05) with Berberol^®^ K, compared to placebo. There was a decrease of high sensitivity C-reactive protein (−30.8%, *p* < 0.05), and interleukin-6 (−25.0%, *p* < 0.05), with Berberol^®^ K compared to placebo. In conclusion, combining different hypocholesterolemic nutraceutical agents such as *Berberis aristata*, *Silybum marianum* and monacolin K and KA could be effective and safe to obtain a reduction of lipid profile and an improvement of inflammatory parameters.

## 1. Introduction

Cardiovascular disease, and in particular coronary heart disease (CHD), is the main cause of mortality in developed countries [1]. The atherosclerotic process plays a central role in the development of cardiovascular diseases; atherosclerosis is the leading cause of heart attacks, stroke, and peripheral vascular disease. In atherosclerosis development, cholesterol, and in particular low density lipoprotein (LDL) cholesterol, plays a main role: usually, atherosclerosis begins with an endothelial damage caused by high blood pressure, smoke, or high cholesterol. At this stage, LDL cholesterol enters the wall of the artery, and this causes white blood cells to stream in to digest the LDL. Over years, this process generates atherosclerotic plaque, until artery obstruction. For this reason, lowering cholesterol levels is of primary importance, and to reach this aim, several molecules have become available in the latest years. The most used hypocholesterolemic agents are statins, or 3-hydroxy-3-methyl-glutaryl-coenzyme A (HMG-CoA) reductase inhibitors. They are effective in reducing cholesterol [2], but they also have some potential adverse effects, including muscle pain with creatine phosphokinase (CPK) elevation, fatigue and weakness, and liver transaminases increase [3]. To avoid statins’ side effects, often physicians consider the use of hypocholesterolemic nutraceutical agents in subjects at low cardiovascular risk [4,5]. Among nutraceuticals, the combination of *Berberis aristata* 588 mg and *Silybum marianum* 105 mg largely proved to be effective and safe [6,7,8].

Recently, a new nutraceutical (Berberol^®^ K) containing berberine 500 mg from *Berberis aristata*, silymarin 105 mg from *Silybum marianum*, and monacolin K and KA 10 mg became available. This combination was marketed with the aim to use the different mechanisms of actions of each compound to have a synergic effect on lipid profile. In particular, *Berberis aristata* up-regulates LDL-receptor (LDL-R) expression independent of sterol regulatory element binding proteins, but dependent on extracellular signal-regulated kinases (ERK) and c-Jun N-terminal kinase (JNK) [9]. *Silybum marianum*, instead, is a potential P-gycoprotein (P-gp) inhibitor, and it is traditionally used as liver protectant [10,11]. Monacolins are a family of substances obtained from the fermentation of rice by the yeast, *Monascus purpureus*. Monacolins act as reversible inhibitors of the HMG-CoA reductase. Among monacolins, monacolin K, also known as mevinolin or lovastatin, and monacolin KA are normally the most prominent monacolins found in fermented red rice extracts [12]. In this context, we planned to carry out a randomized study to evaluate the efficacy and safety of a nutraceutical agent containing berberine, silymarin and monacolin K and KA in a sample of Caucasian patients at low cardiovascular risk. The primary outcome was to evaluate the effects of this nutraceutical combination on lipid profile; secondary outcomes considered the changes on some inflammatory biomarkers.

## 2. Results

### 2.1. Study Sample

A total of 143 patients were enrolled in the trial. Of these, 73 were randomized to Berberol^®^ K and 70 to placebo. One hundred and thirty-nine subjects completed the study; there were four patients who did not complete the study and the reasons for premature withdrawal included non-compliance to treatment or lost to follow-up. The characteristics of the patient population at study entry are shown in Table 1 and Table 2.

No significant changes in body weight, body mass index (BMI) or circumferences were observed in either group (Table 1).

### 2.2. Glycemia, Insulin and the Homeostasis Model Assessment (HOMA) Index

We recorded a statistically significant reduction of fasting plasma glucose (FPG) with Berberol^®^ K compared to placebo (*p* < 0.05). Moreover, we recorded an increase of fasting plasma insulin (FPI) with Berberol^®^ K both compared to baseline and to placebo (*p* < 0.05 for both). Accordingly, the HOMA index obtained after treatment with Berberol^®^ K was lower than the one in the placebo group (*p* < 0.05) (Table 2).

### 2.3. Lipid Profile

No variations of lipid profile were observed with placebo, while there was a statistically significant decrease of total cholesterol (TC), triglycerides (Tg), and low density lipoprotein cholesterol (LDL-C) with Berberol^®^ K, both compared to baseline, and to placebo (*p* < 0.05 for both) (Table 2).

### 2.4. Inflammatory Markers

There was a decrease of high sensitivity C-reactive protein (Hs-CRP), interleukin-6 (IL-6) and tumor necrosis factor-α (TNF-α) with Berberol^®^ K, but not with placebo, compared to baseline (*p* < 0.05 for all); moreover, Hs-CRP and IL-6 obtained with Berberol^®^ K were lower than the ones recorded with placebo (Table 2).

### 2.5. Compliance and Safety

Patients’ compliance to treatment was about 93%. No patients reported serious adverse events. No patients experienced musculoskeletal system disorders, such as myopathy or hepatotoxicity. We did not record any elevation of transaminases (alanine aminotransferase (ALT), aspartate aminotransferase (AST), or CPK) (Table 2).

## 3. Discussion

In the current study, we tested the anti-hyperlipidemic effect of a combination of *Berberis aristata* combined with *Silybum marianum*, and monacolins K and KA, obtaining a LDL-C reduction of about 31.6% compared to baseline.

In previous studies where we tested a nutraceutical combination containing *Berberis aristata* combined with *Silybum marianum*, we reached a LDL reduction of about 24%, and also a decrease of TC and Tg, similar to the current results [6,7]. When we previously tested a nutraceutical combination of monacolins + Coenzime-Q10, instead, we observed a reduction in total cholesterol and LDL-C of about 22%, but no effects on HDL-C and Tg were recorded [13]. Thus, we can assume that, as expected, combining *Berberis aristata* and *Silybum marianum*, with monacolins K and KA better improved LDL-C, while the reduction in Tg could be due to *Berberis aristata* and *Silybum marianum*.

Our research group already demonstrated that *Berberis aristata* combined with *Silybum marianum* were well-tolerated and effective in reducing cholesterolemia and estimated risk of cardiovascular disease [6,7,8]. Regarding the effect on glycemia and insulin, the reductions of glycemia, and the increase of insulinemia that we recorded were similar to our previous studies on *Berberis aristata/Silybum marianum* [6,7], suggesting a neutral effect of monacolin on these parameters. Furthermore, the nutraceutical combination also gave a reduction of inflammatory markers including Hs-CRP, tumor necrosis factor-α (TNF-α) and IL-6; this was expected considering the improvement of lipid profile. Tumor necrosis factor-α is a macrophage-derived inflammatory factor; it alters insulin signaling in cultured cells and in vivo [14], and it has been reported that chronic exposure of cells or whole animals to TNF-α induces insulin resistance [15]. Regarding Hs-CRP, it has been shown to independently predict myocardial infarction, stroke and peripheral artery disease [16]. Finally, IL-6, together with TNF-α, and Hs-CRP, have pro-atherogenic actions, because they increase vascular inflammation [17].

Despite the better effects on lipid profile, we did not observe a major incidence of adverse events, in line with our previous studies [6,7,8], confirming the good tolerability of this nutraceutical combination.

Of course, our study has some limitations, such as the short duration of the trial; moreover, we evaluated only some inflammatory markers, focusing our attention on a few of them. Finally, we did not observe whether the effect of nutraceutical agents was reversible after the interruption of the study.

## 4. Materials and Methods

### 4.1. Study Design

This 3-months, double-blind, randomized, placebo-controlled, clinical trial was conducted at the Department of Internal Medicine and Therapeutics, University of Pavia (Pavia, Italy), among patients attending the Centre for the Treatment of Diabetes and Metabolic Diseases.

The study protocol was approved by the local institutional ethical committee and was conducted in accordance with the 1994 Declaration of Helsinki [18], and its amendments and the Code of Good Clinical Practice. All patients provided written informed consent to participate in this study after a full explanation of the study had been given.

### 4.2. Patients

Caucasian patients aged ≥18 of either sex were eligible for inclusion in the study if they were at low cardiovascular risk according to the Framingham Risk Score [19]; and if they had hypercholesterolemia according to National Cholesterol Education Program (NCEP) Expert Panel on Detection, Evaluation, and Treatment of High Blood Cholesterol in Adults (ATP III) criteria [20] (TC between 200 and 240 mg/dL), and with Tg < 400 mg/dL. They were overweight (BMI 25.0–29.9 kg/m^2^) [21], and also normotensive subjects according to the World Health Organization criteria (systolic blood pressure (SBP) < 140 mmHg and diastolic blood pressure (DBP) < 90 mmHg) [22]. Furthermore, they were non-smokers.

Suitable patients, identified from a review of case notes and/or computerized clinic registers, were contacted by the investigators in person or by telephone.

Patients were excluded if they had secondary dyslipidemia or type 2 diabetes mellitus; impaired hepatic function (defined as a plasma aminotransferase and/or gamma-glutamil transpeptidase (γ-GT) level higher than the upper limit of normal (ULN) for age and sex); impaired renal function (defined as a serum creatinine level higher than the ULN for age and sex); endocrine disorders, or gastrointestinal disorders; current or previous evidence of ischemic heart disease, heart failure, or stroke; weight change of >3 kg during the preceding 3 months; malignancy; and significant neurological or psychiatric disturbances, including alcohol or drug abuse. Excluded medications (within the previous 3 months) were anorectic agents, laxatives, β-agonists (other than inhalers), diuretics, cyproheptadine, anti-depressants, anti-serotoninergics, phenothiazines, barbiturates, oral corticosteroids, and anti-psychotics. Women who were pregnant or breastfeeding or of childbearing potential and not taking adequate contraceptive precautions were also excluded.

### 4.3. Diet and Physical Activity

All patients were already following an adequate diet and practicing physical activity. The controlled-energy diet (~600 kcal daily deficit) was based on NCEP-ATP III recommendations [20], that contained 50% of calories from carbohydrates, 30% from fat (<7% saturated, up to 10% polyunsaturated, and up to 20% monounsaturated), and 20% from proteins, with a maximum cholesterol content of 300 mg/day, and 35 g/day of fiber. Standard diet advice was given by a dietitian and/or specialist physician. Individuals were also encouraged to increase their physical activity and we standardized the same physical aerobics exercise program by riding a stationary bicycle for 20 to 30 min, three to four times per week. Subjects were instructed to fill diaries daily about food and physical activity and to bring them to the Investigators to assess their compliance to lifestyle.

### 4.4. Treatment

**Figure 1:**
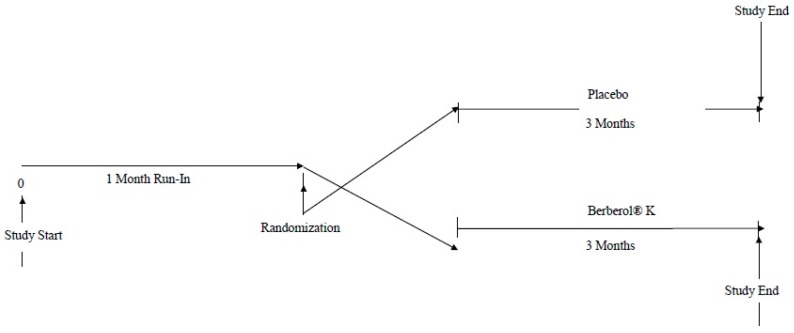
Study design.

After a one-month run-in period during which diet was standardized, patients were randomized to take placebo or Berberol^®^ K for 3 months, in a randomized, double-blind, placebo-controlled design (Figure 1). Berberol^®^ K and placebo were self-administered once a day, one tablet during the dinner. Both Berberol^®^ K and placebo were supplied as identical, opaque, tablets in coded bottles to ensure the blind status of the study. The weight of both tablets was similar, about 1.1 g each one.

Berberol^®^ K is a nutraceutical association in tablet form (traded in Italy by PharmExtracta, Pontenure, Italy) containing 500 mg/tablet of berberine from *Berberis aristata* (extract titration: 96%), 105 mg/tablet of silymarin from *Silybum marianum* (extract titration >60%) and 10 mg/tablet of monacolins (K + KA) from MP-K20^®^, a highly standardized *Monascus purpureus* fermented rice (extract titration: 20%). The product, in agreement with the Italian law number 169/2004, has been notified to the Minister of Health in 2015 (Registration number: 77055) and registered as a food supplement with all its actives (*Berberis aristata*, *Silybum marianum* and *Monascus purpureus* fermented rice standardized extracts) being admitted as nutraceuticals and its excipients all food grade.

Randomization was done using a drawing of envelopes containing randomization codes prepared by a statistician. Medication compliance was assessed by counting the number of pills returned at the time of specified clinic visits. Throughout the study, we instructed patients to take their first dose of the new medication on the day after they were given the study medication. At the same time, all unused medication was retrieved for inventory. All medications were provided free of charge.

### 4.5. Assessments

Before starting the study, all patients underwent an initial screening assessment that included medical history; a physical examination; vital signs (blood pressure and heart rate); a 12-lead electrocardiogram; measurements of a 12-lead electrocardiogram; measurements of height and body weight; calculation of BMI; abdominal circumference (Abd. Cir.); waist circumference (Waist Cir.) and hip circumference (Hip Cir.); assessment of FPG, FPI, the HOMA index, TC, LDL-C, high density lipoprotein-cholesterol (HDL-C), Tg, Hs-CRP, and TNF-α IL-6. All parameters were assessed at baseline and after 3 months from the study start.

For a description of how various parameters assays were performed, please see our previous papers [23,24].

### 4.6. Safety Measurements

Treatment tolerability was assessed at each study visit using an accurate interview of patients by the investigators, and comparisons of clinical and laboratory values with baseline levels. Safety monitoring included physical examination, vital sign assessment, weight, electrocardiogram, adverse events, and laboratory tests. Liver and muscle function were evaluated by measurement of transaminases (aspartate aminotransferase (AST), alanine aminotransferase (ALT), and CPK), and all adverse events were recorded.

### 4.7. Statistical Analysis

An intention-to-treat (ITT) analysis was conducted in patients who had received a ≥1 dose of the study medication and had a subsequent efficacy observation. Considering a difference of at least 10% compared to the baseline and an alpha error of 0.05 as clinically significant, the actual sample size was adequate to obtain a power higher than 0.80 to detect a significant between-group difference in variables related to lipid profile. Patients were included in the tolerability analysis if they had received ≥1 dose of trial medication after randomization and had undergone a subsequent tolerability observation. The null hypothesis that the expected mean TC, LDL-C, HDL-C, and Tg change from the end of the study did not differ significantly between placebo, and Berberol^®^ K was tested using analysis of variance and analysis of covariance (ANCOVA) models [25]. Similar analyses were applied to the other variables. The statistical significance of the independent effects of treatments on the other variables was determined using ANCOVA. A 1-sample *t* test was used to compare values obtained before and after treatment administration; 2-sample *t* tests were used for between-group comparisons. Statistical analysis of data was performed using the Statistical Package for Social Sciences software version 11.0 (SPSS Inc., Chicago, IL, USA). Data are presented as mean (SD). For all statistical analyses, *p* < 0.05 was considered statistically significant.

## 5. Conclusions

The results of this study suggested that combining different hypocholesterolemic nutraceutical agents such as *Berberis aristata*, *Silybum marianum* and monacolin K and KA could be effective and safe to obtain a reduction of LDL-C and an improvement of inflammatory parameters in subjects at low risk of cardiovascular disease. The reduction of Tg, instead, is probably due only to *Berberis aristata* and *Silybum marianum*.

## Figures and Tables

**Table 1:** Demographic and anthropometric characteristics with Berberol^®^ K and placebo.

Parameters	Berberol^®^ K (Berberine, Silymarin and Monacolins (K + KA)		Placebo (Dummy Pill Containing Lactose)	
	Baseline	3 Months	Baseline	3 Months
*n*	73	71	70	68
Sex (M/F)	35/38	35/36	35/35	33/35
Age (years)	50.2 ± 6.2	-	47.4 ± 5.3	-
Height (m)	1.68 ± 0.04	-	1.69 ± 0.05	-
Weight (kg)	78.7 ± 9.2	78.4 ± 8.9	77.8 ± 8.8	77.2 ± 8.2
BMI (kg/m^2^)	27.9 ± 1.1	27.8 ± 1.0	27.2 ± 0.8	26.9 ± 0.7
Abd. Cir. (cm)	90.5 ± 3.1	89.7 ± 2.9	89.4 ± 2.8	89.0 ± 2.6
Waist Cir. (cm)	87.4 ± 2.5	87.1 ± 2.3	86.8 ± 2.3	86.2 ± 2.1
Hip Cir. (cm)	99.8 ± 3.8	99.2 ± 3.3	99.1 ± 3.5	98.2 ± 3.1

Data are expressed as mean ± standard deviations (SD); Abd. Cir.: abdominal circumference; Waist Cir.: waist circumference; Hip Cir.: hip circumference; BMI: body mass index.

**Table 2:** Variation of lipid profile, inflammatory markers and safety parameters with Berberol^®^ K and placebo.

Parameters	Berberol^®^ K (Berberine, Silymarin and monacolins (K + KA))		Placebo (Dummy Pill Containing Lactose)		Differences		
	Baseline	3 Months	Baseline	3 Months	Berberol^®^ K vs. Baseline (%)	Berberol^®^ K vs. Placebo (Δ)	Berberol^®^ K vs. Placebo (%)
*n*	73	71	70	68			
FPG (mg/dL)	88.8 ± 7.2	82.6 ± 6.7 ^†^	90.2 ± 7.9	94.1 ± 8.5	−7.0	−11.5	−12.2
FPI (µUI/mL)	9.4 ± 5.7	10.1 ± 6.0 *^,†^	9.3 ± 5.3	9.1 ± 5.1	+6.9	+1.0	+9.9
HOMA index	2.06 ± 0.42	2.05 ± 0.40^†^	2.07 ± 0.44	2.11 ± 0.48	−0.49	−0.06	−2.85
TC (mg/dL)	222.6 ± 33.5	171.6 ± 20.6 *^,†^	219.1 ± 30.9	215.7 ± 31.8	−22.9	−44.1	−20.5
LDL-C (mg/dL)	155.6 ± 13.8	106.5 ± 11.4 *^,†^	151.1 ± 12.9	147.6 ± 13.7	−31.6	−41.1	−27.8
HDL-C (mg/dL)	42.1 ± 4.2	45.4 ± 4.9	43.9 ± 5.0	44.2 ± 5.2	+7.3	+1.2	+2.7
Tg (mg/dL)	124.5 ± 37.4	98.4 ± 22.5 *^,†^	120.8 ± 36.8	119.5 ± 35.9	−21.0	−21.1	−17.7
AST (U/L)	19.4 ± 10.4	19.1 ± 10.1	19.0 ± 10.1	19.8 ± 10.4	−1.5	−0.7	−3.5
ALT (U/L)	22.5 ± 12.4	19.2 ± 10.2	24.7 ± 13.8	25.3 ± 14.2	−14.7	−6.1	−24.1
CPK (U/L)	162.4 ± 47.3	154.2 ± 42.8	158.6 ± 45.2	165.3 ± 47.2	−5.1	−11.1	−6.8
Hs-CRP (mg/L)	1.3 ± 0.8	0.9 ± 0.6 *^,†^	1.4 ± 0.9	1.3 ± 0.8	−30.8	−0.4	−30.8
TNF-α (ng/mL)	1.4 ± 0.9	1.0 ± 0.5 *	1.3 ± 0.8	1.2 ± 0.6	−28.6	−0.2	−16.7
IL-6 (pg/mL)	1.4 ± 0.7	0.9 ± 0.3 *^,†^	1.4 ± 0.7	1.2 ± 0.5	−35.7	−0.3	−25.0

Data are expressed as mean ± standard deviations (SD); * *p* < 0.05 vs. baseline; ^†^ *p* < 0.05 vs. placebo; FPG: fasting plasma glucose; FPI: fasting plasma insulin; TC: total cholesterol; LDL-C: low density lipoprotein-cholesterol; HDL-C: high density lipoprotein-cholesterol; Tg: triglycerides; AST: alanine aminotransferase; AST: aspartate aminotransferase; CPK: creatinine phosphokinase; Hs-CRP: high-sensitivity C-reactive protein; TNF-α: tumor necrosis factor-α; IL-6: interleukin-6; HOMA: homeostasis model assessment.

## Abbreviations

Abd. Cir.	abdominal circumference
Waist Cir.	waist circumference
Hip Cir.	hip circumference
BMI	body mass index
FPG	fasting plasma glucose
FPI	fasting plasma insulin
TC	total cholesterol
LDL-C	low density lipoprotein-cholesterol
HDL-C	high density lipoprotein-cholesterol
Tg	triglycerides
ALT	alanine aminotransferase
AST	aspartate aminotransferase
CPK	creatinine phosphokinase
Hs-CRP	high-sensitivity C-reactive protein
TNF-α	tumor necrosis factor-α
IL-6	interleukin-6
CHD	coronary heart disease

## References

[1] Muller-Nordhorn J., Binting S., Roll S., Willich S.N. (2008). An update on regional variation in cardiovascular mortality within Europe. Eur. Heart J..

[2] Vijan S., Hayward R.A., American College of Physicians (2004). Pharmacologic lipid-lowering therapy in type 2 diabetes mellitus: Background paper for the American College of Physicians. Ann. Intern. Med..

[3] McClure D.L., Valuck R.J., Glanz M., Hokanson J.E. (2007). Systematic review and meta-analysis of clinically relevant adverse events from HMG CoA reductase inhibitor trials worldwide from 1982 to present. Pharmacoepidemiol. Drug Saf..

[4] Derosa G., Maffioli P. (2015). Nutraceuticals for the treatment of metabolic diseases: Evidence from clinical practice. Expert Rev. Endocrinol. Metab..

[5] Scicchitano P., Cameli M., Maiello M., Modesti P.A., Muiesan M.L., Novo S., Palmiero P., Saba P.S., Pedrinelli R., Ciccone M.M. (2014). Nutraceuticals and dyslipidaemia: Beyond the common therapeutics. J. Funct. Foods.

[6] Derosa G., Bonaventura A., Bianchi L., Romano D., D’Angelo A., Fogari E., Maffioli P. (2013). *Berberis aristata/Silybum marianum* fixed combination on lipid profile and insulin secretion in dyslipidemic patients. Expert Opin. Biol. Ther..

[7] Derosa G., Bonaventura A., Bianchi L., Romano D., D’Angelo A., Fogari E., Maffioli P. (2013). Effects of *Berberis aristata/Silybum marianum* association on metabolic parameters and adipocytokines in overweight dyslipidemic patients. J. Biol. Regul. Homeost. Agents.

[8] Derosa G., Romano D., D’Angelo A., Maffioli P. (2015). *Berberis aristata/Silybum marianum* fixed combination (Berberol^®^) effects on lipid profile in dyslipidemic patients intolerant to statins at high dosages: A randomized, placebo-controlled, clinical trial. Phytomedicine.

[9] Kong W., Wei J., Abidi P., Lin M., Inaba S., Li C., Wang Y., Wang Z., Si S., Pan H. (2004). Berberine is a novel cholesterol-lowering drug working through a unique mechanism distinct from statins. Nat. Med..

[10] Luper S. (1998). A review of plants used in the treatment of liver disease: Part 1. Altern. Med. Rev..

[11] Kidd P., Head K. (2005). A review of the bioavailability and clinical efficacy of milk thistle phytosome: A silybin-phosphatidylcholine complex (Siliphos). Altern. Med. Rev..

[12] Nannoni G., Alì A., di Pierro F. (2015). Development of a new highly standardized and granulated extract from Monascus purpureus with a high content of Monacolin K and KA and free of inactive secondary monacolins and citrinin. Nutrafoods.

[13] Cicero A.F., Derosa G., Parini A., Maffioli P., D’Addato S., Reggi A., Giovannini M., Borghi C. (2013). Red yeast rice improves lipid pattern, high-sensitivity C-reactive protein, and vascular remodeling parameters in moderately hypercholesterolemic Italian subjects. Nutr. Res..

[14] Hotamisligil G.S. (1999). The role of TNF-α and TNF receptors in obesity and insulin resistance. J. Intern. Med..

[15] Uysal K.T., Wiesbrock S.M., Marino M.W., Hotamisligil G.S. (1997). Protection from obesity-induced insulin resistance in mice lacking TNF-α function. Nature.

[16] Zwacka T.P., Hornbach V., Torzewski J. (2001). C-reactive protein-mediated lipoprotein uptake by macrophages. Circulation.

[17] Matsuzawa Y. (2006). Therapy insight: Adipocytokines in metabolic syndrome and related cardiovascular disease. Nat. Clin. Pract. Cardiovasc. Med..

[18] The Council for International Organisation of Medical Sciences Proposed International Guidelines for Biomedical Research Involving Human Subjects. http://www.cioms.ch/publications/layout_guide2002.pdf.

[19] D’Agostino R.B., Vasan R.S., Pencina M.J., Wolf P.A., Cobain M., Massaro J.M., Kannel W.B. (2008). General cardiovascular risk profile for use in primary care: The Framingham Heart Study. Circulation.

[20] Expert Panel on Detection, Evaluation, and Treatment of High Blood Cholesterol in Adults (2001). Executive summary of the third report of the national cholesterol education program (NCEP) expert panel on detection, evaluation and treatment of high blood cholesterol in adults (Adult Treatment Panel III). JAMA.

[21] World Health Organization Obesity: Preventing and Managing the Global Epidemic. http://www.who.int/nutrition/publications/obesity/WHO_TRS_894/en/.

[22] World Health Organization—International Society of Hypertension (1999). Guidelines for the management of hypertension. Guidelines Subcommittee. J. Hypertens..

[23] Derosa G., Cicero A.F., Fogari E., D’Angelo A., Bonaventura A., Romano D., Maffioli P. (2012). Effects of n-3 PUFAs on postprandial variation of metalloproteinases, and inflammatory and insulin resistance parameters in dyslipidemic patients: Evaluation with euglycemic clamp and oral fat load. J. Clin. Lipidol..

[24] Derosa G., Mugellini A., Pesce R.M., D’Angelo A., Maffioli P. (2016). Barnidipine compared to lercanidipine in addition to losartan on endothelial damage and oxidative stress parameters in patients with hypertension and type 2 diabetes mellitus. BMC Cardiovasc. Disord..

[25] Winer B.J. (1971). Statistical Principles in Experimental Design.

